# Exploring the Potential Genetic Heterogeneity in the Incidence of Hoof Disorders in Austrian Fleckvieh and Braunvieh Cattle

**DOI:** 10.3389/fgene.2020.577116

**Published:** 2020-11-17

**Authors:** Barbara Kosińska-Selbi, Tomasz Suchocki, Christa Egger-Danner, Hermann Schwarzenbacher, Magdalena Frąszczak, Joanna Szyda

**Affiliations:** ^1^Biostatistic Group, Department of Genetics, Wrocław University of Environmental and Life Sciences, Wrocław, Poland; ^2^National Research Institute of Animal Production, Balice, Poland; ^3^ZuchtData EDV-Dienstleistungen GmbH, Vienna, Austria

**Keywords:** Braunvieh, Fleckvieh, genetic heterogeneity, GWAS, health traits, linkage disequilibrium, principal components

## Abstract

Genetic heterogeneity denotes the situation when different genetic architectures underlying diverse populations result in the same phenotype. In this study, we explore the genetic background underlying differences in the incidence of hoof disorders between Braunvieh and Fleckvieh cattle in the context of genetic heterogeneity between the breeds. Despite potentially higher power of testing due to twice as large sample size, none of the SNPs was significantly associated with the total number of hoof disorders in Fleckvieh, while 15 SNPs were significant in Braunvieh. The most promising candidate genes in Braunvieh were as follows: *CBLB* on BTA1, which causes arthritis in rats; *CAV2* on BTA4, which affects skeletal muscles in mice; *PTHLH* on BTA5, which causes disease phenotypes related to the skeleton in humans, mice, and zebrafish; and *SORCS2* on BTA6, which causes decreased susceptibility to injury in mice. Some of the significant SNPs (BTA1, BTA4, BTA5, BTA13, and BTA16) revealed allelic heterogeneity—i.e., different allele frequencies between Fleckvieh and Braunvieh. Some of the significant regions (BTA1, BTA5, BTA13, and BTA16) correlated to inter-breed differences in linkage disequilibrium (LD) structure and may thus represent false-positive heterogeneity. However, positions on BTA6 (*SORCS2*), BTA14, and BTA24 mark Braunvieh-specific regions. We hypothesize that the observed genetic heterogeneity of hoof disorders is a by-product of different selection goals defined for the analyzed breeds—toward dairy production in Braunvieh and toward beef production in Fleckvieh. Based on the current dataset, it is not possible to unequivocally confirm or exclude the hypothesis of genetic heterogeneity in the susceptibility to hoof disorders between Fleckvieh and Braunvieh. The main reason for the problem is that the potential heterogeneity was explored through SNP–phenotype associations and not through causal mutations, due to a limited SNP density offered by the SNP-chip. The rationale against genetic heterogeneity comprises a limited power of detection of true associations as well as differences in the length of LD blocks and in linkage phase between breeds. On the other hand, different selection goals defined for the analyzed breeds accompanied by no systematic, genome-wide differences in LD structure between the breeds favor the heterogeneity hypothesis at some smaller genomic regions.

## Introduction

Genetic heterogeneity denotes the situation when different genetic architectures underlying diverse populations result in the same phenotype. In human genetics, for decades, the concept of genetic heterogeneity has been considered and investigated using genome-wide association studies (GWAS) (Lander and Schork, 1994). One of the most well-known diseases characterized by high degree of genetic heterogeneity is the human autism spectrum disorder (An and Claudianos, 2016). Relatively recently, the concept of genetic heterogeneity has also been considered in the analysis of data from artificially selected plant and livestock species by Bérénos et al. (2015); de los Campos et al. (2015), and Lehermeier et al. (2015). In plants and livestock, an important source of genetic heterogeneity may be their complex population structure, which is typically composed of divergently selected breeds exhibiting high variation in allele frequencies and linkage disequilibrium (LD) patterns (de los Campos and Sorensen, 2014). In cattle, hoof disorders are novel traits represented by a group of different phenotypes varying from binary, directly assessed disease diagnoses, such as a sole ulcer, to composite traits scored on a categorical basis, e.g., a locomotion score. Due to their impact on welfare, productivity, and fertility (Heringstad et al., 2018), the traits rapidly gain importance in cattle breeding schemes. Technically, a common feature of this phenotypic group is a relatively poor definition of traits and lack of routine recording, resulting in a large number of relatively small datasets scattered across various populations. Those features not only cause low power of detection of significant gene (or SNP)–phenotype associations, resulting in a low reproducibility of results (Heringstad et al., 2018) but also imply a potential heterogeneity in the genetic determination of phenotypes due to differences in selection schemes. Underlying differences in LD and allele frequencies between populations cloud proof of potential heterogeneity (Veturi et al., 2019). In this study, we explore the genetic background underlying differences in the incidence of the number of hoof disorders between Austrian breeds Braunvieh and Fleckvieh cattle in the context of genetic heterogeneity of the breeds.

## Materials and Methods

### Dataset

**FIGURE 1:**
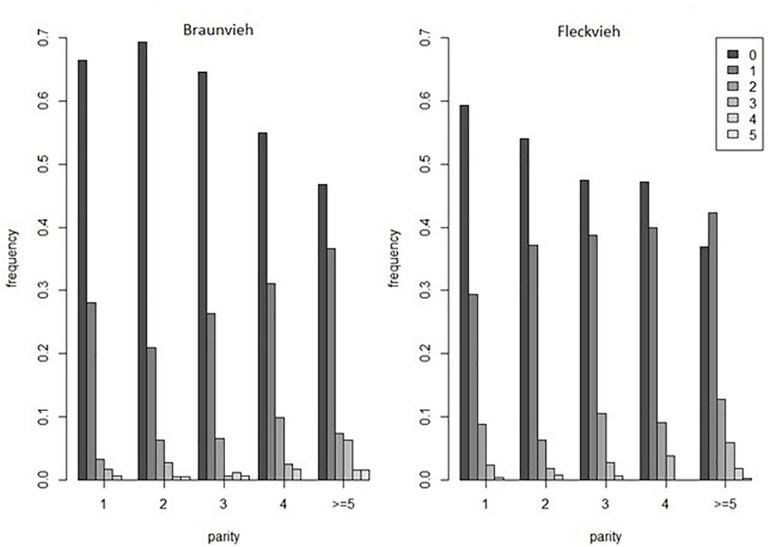
The distribution of the number of hoof disorders in Braunvieh and Fleckvieh.

The analyzed dataset was collected within the frame of the Efficient Cow project and comprised scores of hoof and leg disorders in Austrian 985 Braunvieh and 1999 Fleckvieh cows. In particular, the analyzed phenotype comprised the total number of hoof disorders scored by claw trimmers until the 100th day of lactation. The considered disorders comprised digital dermatitis, double sole, heel horn erosion, interdigital hyperplasia, sole hemorrhage, sole ulcer, and white line separation. In both breeds, the number of disorders varied between none and five, but the distributions differed. The fraction of diseased cows was higher in Fleckvieh (Figure 1). The cows were genotyped with the GeneSeek^®^ Genomic Profiler^TM^ HD panel consisting of 76,934 SNPs. After quality control based on a minor allele frequency (<0.01) and a per-individual call rate (<99%), for further analysis, 74,762 SNPs remained.

### GWAS and Functional Gene Annotation

The GWAS was performed separately for each breed by applying a single-SNP mixed linear model implemented in the GCTA software (Yang et al., 2011) with pseudophenotypes, expressed by cows’ breeding values, estimated by Suchocki et al. (2020):

$$\text{y}=\text{X}\beta +{\text{Z}}_{u}\text{u}+{\text{Z}}_{v}\text{v}+{\text{Z}}_{p}\text{p}+\varepsilon ,$$

where ***y*** is a vector of total number of hoof disorders scored until the 100th day of lactation; **β** is a vector of fixed effects comprising a general mean, breed, parity, calving year-season, percent of non-Holstein-Friesian genes, and a hoof status recording code; ***u*** is a random additive polygenic effect of a cow representing her breeding value; ***v*** is a random veterinarian effect; ***p*** is a random permanent environmental effect; and **ε** is a vector of random residuals. It was assumed that $u∼N\left(0,A\sigma_{u}^{2}\right)$, $v∼N\left(0,I_{v}\sigma_{v}^{2}\right)$,$p∼N\left(0,I_{p}\sigma_{p}^{2}\right)$, and $\varepsilon ∼N\left(0,I_{\varepsilon }\sigma_{\varepsilon }^{2}\right)$, where ***A*** is an additive relationship matrix among individuals calculated based on pedigree; $\sigma_{u}^{2}$, $\sigma_{v}^{2}$, $\sigma_{p}^{2},$ and $\sigma_{\varepsilon }^{2}$ are variances for additive polygenic, veterinarian, permanent environmental, and residual effects, respectively; and ***I_v_***, ***I_p_***, and ***I_ε_*** are identity matrices. Then, the single-SNP GWAS was based on the following model:

u=μ+Xb+Zg+e,

where ***u*** is a vector of breeding values estimated as shown above; **μ** is a general mean; ***b*** is a fixed additive effect of a single SNP; ***X*** is a corresponding design matrix coded as 0, 1, or 2 for a homozygous, heterozygous, and the other homozygous genotype respectively; $g∼N\left(0,G\sigma_{g}^{2}\right)$ is a random additive polygenic effect with the genomic covariance matrix between cows (***G***) calculated based on SNP genotypes (Yang et al., 2011); ***Z*** is an incidence matrix for ***g***; and $e∼N\left(0,I\sigma_{e}^{2}\right)$ is a residual. The null hypothesis of ***b*** = 0 was tested using the Likelihood Ratio Test with the asymptotic large sample $\chi_{1}^{2}$ distribution (as implemented in the GCTA). The resulting nominal *P-*values were transformed into false discovery rates (FDRs) (Benjamini and Hochberg, 1995) to account for multiple testing. Functions of genes, which are marked by significant SNPs, were ascertained based on the following resources: the Mouse Genome Informatics phenotype database,^1^ the International Mouse Phenotyping Consortium database,^2^ the Zebrafish Information Network database,^3^ the human Rare Diseases and Orphan Drugs database,^4^ and the Deciphering Developmental Disorders database,^5^ accessed through the Ensembl.

### Analysis of Allelic Heterogeneity

For each non-overlapping window of 50 neighboring SNPs, genomic relationship matrices between cows were calculated, which were then decomposed into principal components, using the PCA subroutine implemented in the GCTA (Price et al., 2006). Further on, for each of the windows, differences between the breeds in a 10-dimensional space defined by the first 10 eigenvectors (**ε**_1_,**ε**_2_,…,**ε**_10_) were quantified using the Mahalanobis distance: $D_{M}=\sqrt{d^{\prime }V^{-1}d}$, with $d=\left[{\bar{\varepsilon }}_{1B}-{\bar{\varepsilon }}_{1F},{\bar{\varepsilon }}_{2B}-{\bar{\varepsilon }}_{2F},\ldots ,{\bar{\varepsilon }}_{10B}-{\bar{\varepsilon }}_{10F}\right]$ containing differences between averaged eigenvectors for Braunvieh (subscript *B*) and Fleckvieh (subscript *F*) and *V* representing the pooled covariance matrix of **ε**_1_ and **ε**_2_. The Hotelling test: $T=\frac{n_{B}n_{F}}{n_{B}+n_{F}}\cdot \frac{n_{B}+n_{F}-11}{10\left(n_{B}+2n_{F}-2\right)}\cdot d^{\prime }V^{-1}d∼F_{10,n_{B}+n_{F}-11}$ was used to test the null hypothesis of no differences between Braunvieh and Fleckvieh, where *n*_*x*_ is the number of cows representing breed *x* (Goodpaster and Kennedy, 2011). In order to account for multiple testing, the resulting nominal *P-*values were transformed into FDRs.

The allelic heterogeneity between breeds was tested by calculating the ratio of minor allele frequencies in Fleckvieh (*MAF*_*F*_) and Braunvieh (*MAF*_*B*_) at significant SNP positions. For hypotheses testing, the natural logarithm of the ratio was used: $ln\left(\frac{MAF_{F}}{MAF_{B}}\right)∼N\left(0,1\right)$.

### Analysis of Local LD Patterns

Differences in LD patterns between breeds were assessed based on the comparison of LD matrices constructed for non-overlapping windows of 50 neighboring SNPs. LD between the pairs of linked SNPs was quantified using Beagle 4.1 (Browning and Browning, 2016), separately for each breed, by the *r*^2^ coefficient given by $\frac{{\left(p_{11}p_{22}-p_{12}p_{21}\right)}^{2}}{p_{1.}\left(1-p_{1.}\right)p_{.1}\left(1-p_{.1}\right)}$, with *p*_*ij*_ corresponding to the frequency of a two-SNP haplotype *i*,*j* ∈ {11,12,21,22}, and *p*_*1.*_ and *p*_*.1*_ representing the frequency of an alternative allele respectively for the 1st and the 2nd SNP (VanLiere and Rosenberg, 2008). Eigenvectors, which correspond to these LD matrices, were computed separately for each breed. Inter-breed differences in local LD were then quantified by:

$$S=2\sum_{i=1}^{50}\left[{\left(v_{iBB}-v_{iFB}\right)}^{2}+{\left(v_{iBF}-v_{FF}\right)}^{2}\right],$$

where *v*_*ijk*_ corresponds the *i*-th element of the 1st principal component vector calculated as the product of the LD matrix of the *j*-th breed (subscript *B* for BSW or *F* for FLV) and the 1st eigenvector of the *k*-th breed. Following Garcia (2012), *S* quantifies differences in the variability of LD between two populations. Furthermore, the genome-wide pattern of LD decay with physical distance between pairwise SNPs was binned into nine intervals (0–25, 25–50, 50–75, 75–120, 120–200, 200–500, 500–1500, 1500–3000, and 3000–5000 kbp).

## Results

### Heterogeneity in Association Signals

**FIGURE 2:**
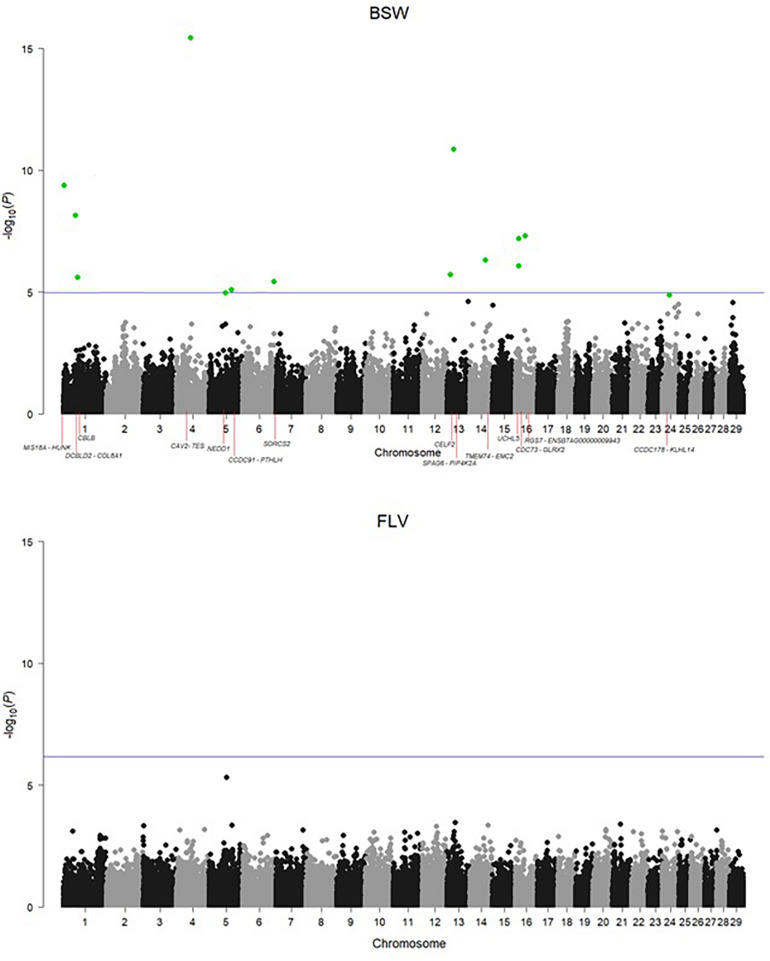
Manhattan plots for the incidence of hoof disorders in Braunvieh and Fleckvieh. The horizontal line corresponds to an FDR of 10%. The significant SNPs were highlighted in green.

Adapting the FDR threshold of 10%, despite potentially higher power of testing due to twice as large sample size, none of the SNPs was significantly associated with the number of hoof disorders in Fleckvieh, while 15 SNPs were significant in Braunvieh (Figure 2). One of the three significant SNPs from BTA1 is located 285,955 bp upstream of *CBLB* gene, known to cause arthritis in rats. The same SNP is located within a region of a QTL for hindquarter proportions. The two most significant SNPs were located on BTA4. Both were intergenic, but their closest downstream gene encodes caveolin 2 protein, which in the mouse is known to effect skeletal muscles. A SNP on BTA5 falls within four QTL regions responsible for rump conformation traits. One of the most interesting significant annotations points out at another SNP on BTA5, which is located 76,362 bp downstream of *PTHLH*. In humans and mice, this gene causes multiple disease phenotypes related to the skeleton. In zebrafish, mutations of this gene result in decreased bone mineralization, in humans—in brachydactyly as well as in numerous bone and calcium related disease phenotypes in the mouse including decreased length of long bones, premature bone ossification, and increased osteocyte apoptosis. Another interesting significant annotation points out at the intron of *SORCS2* gene on BTA6, which was assigned to decreased susceptibility to injury in the mouse. The effect on muscles, albeit in zebrafish, was assigned to the protein encoded by *PIP4K2A*, which is located close to the significant SNP on BTA13. The same SNP is also located within a QTL region for rump angle. On BTA16, a significant association points out at *ENSBTAG00000009943* involved in inflammatory response. Significant SNPs are summarized in Table 1 except a SNP on BTA6, which could not be placed on the current reference assembly (ARS-UCD1.2).

**TABLE 1:** SNPs significant in BSW, based on FDR ≤ 0.10.

Position^1^ ID	Closest gene(s)	QTL^2^ (ID)	Effect	Increasing allele	FDR for SNP effect	FDR for Mahalanobis distance	*S*	FDR for MAF frequency ratio
1:3,303,269 rs110488513	Intergenic between *MIS18A* and *HUNK*		0.018	A	0.000008	1.0	0.163591	0.391039
1:43,542,488 rs41661497	Intergenic between *DCBLD2* and *COL8A1*		0.012	G	0.000106	1.0	0.152780	0.229225
1:50,767,507 rs110811919	Intergenic upstream of *CBLB*	Hindquarter proportions (7124)	0.007	G	0.015146	1.0	0.152780	0.007376
4:52,028,036 rs110514562	Intergenic between *CAV2* and *TES*		0.029	A	<10^–6^	1.0	0.002094	<10^–7^
4:52,079,221 rs137336750	Intergenic between *CAV2* and *TES*		0.029	G	<10^–6^	1.0	0.002094	<10^–7^
5:61,220,624 rs41590733	Intergenic upstream of *NEDD1*	Rump conformation (3422, 3424, 1563, 20622)	0.010	A	0.052989	1.0	0.005398	0.030671
5:81,769,685 rs109268584	Intergenic between *CCDC91* and *PTHLH*		0.008	A	0.043017	1.0	0.005398	0.008493
6:114,116,280 rs110962969	Intron of *SORCS2*		0.010	C	0.002456	1.0	0.006265	0.381230
13:13,590,662 rs110792762	Intergenic upstream of *CELF2*		0.008	G	0.012588	1.0	0.451395	0.003215
13:23,590,146 rs110989397	Intergenic between *SPAG6* and *PIP4K2A*	Rump angle (3429)	0.019	A	<10^–6^	1.0	0.006076	0.019040
14:55,768,446 rs110534995	Intergenic between *TMEM74* and *EMC2*		0.010	A	0.003962	1.0	0.023641	0.153087
16:12,125,227 rs29024589	Intergenic between *CDC73* and *GLRX2*		0.010	T	0.000676	1.0	0.021476	0.394140
16:12,280,122 rs110843300	Intergenic upstream of *UCHL5*		0.008	G	0.006398	1.0	0.021475	0.398872
16:36,037,389 rs41579631	Intergenic between *RGS7* and *ENSBTAG00000009943*		0.007	G	0.000592	1.0	0.026922	<10^–115^
24:24,273,191 rs136424124	Intergenic between *CCDC178* and *KLHL14*		0.002	A	0.082299	1.0	0.006277	0.111094

*^1^Corresponding to the ARD-UCS1.2 assembly. ^2^QTLdb release 42 (www.animalgenome.org/QTLdb/cattle/).*

There was no correlation between *P*-values observed for SNPs in FLV and BSW, which was estimated to 0.00302 for all SNPs and −0.01494 (−0.08065) for SNPs with 100 smallest *P*-values in BSW (FLV). In addition, breed-specific SNP effect estimates also revealed a very low correlation of 0.02363.

### Heterogeneity in Genetic Structure

**FIGURE 3:**
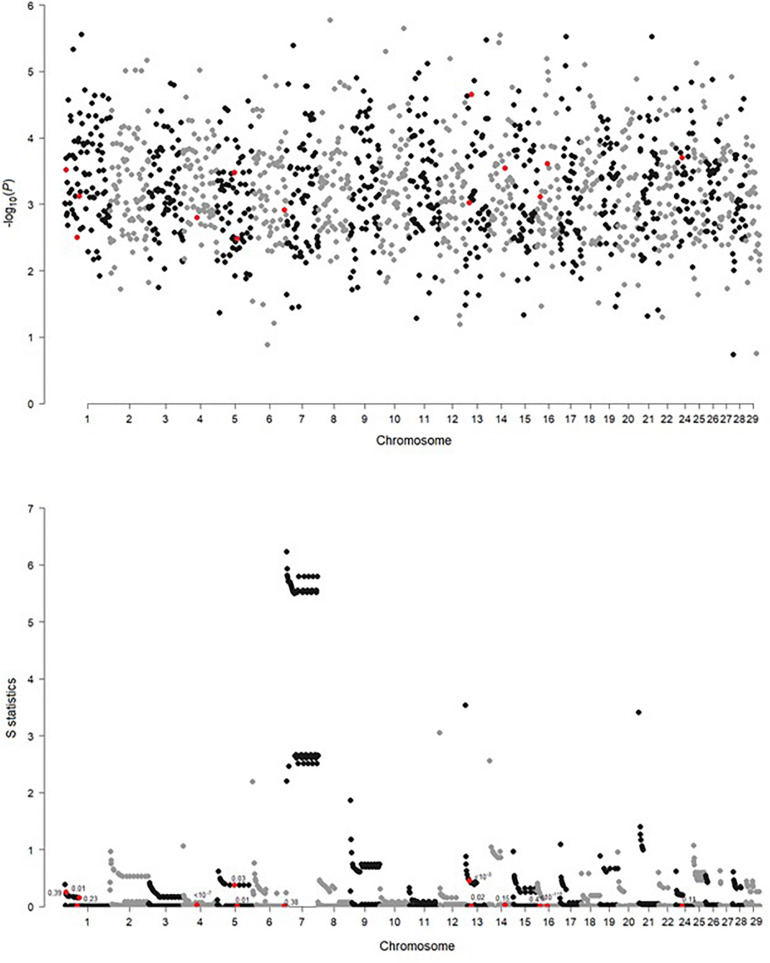
Genome-wide comparison of genetic heterogeneity expressed by significance of the Mahalanobis distance and the *S* statistic along the windows composed of 50 neighboring SNPs. Each point marked in red indicates a window containing the SNP significant in GWAS.

Significance of the Mahalanobis distances (expressing differences in SNP genotype variability between breeds) and values of the *S* statistic (expressing differences in the LD decay pattern between breeds) were visualized in Figure 3. For the Mahalanobis distance, all FDR values at the significant SNP locations are equal to 1, indicating that it was not possible to differentiate between breeds based on SNP genotypes corresponding to the 50-SNP windows. A somewhat different picture emerged when the local inter-breed differences in LD decay were considered. In some, but not all, of the regions, significant SNPs correspond to windows for which a difference in LD structure was indicated by high values of the *S* statistics—rs110488513 and rs41661497 on BTA1 as well as rs110792762 on BTA13. Some other significant SNPs are located in windows adjacent to such windows—rs110811919 on BTA1, as well as rs29024589, rs110843300, and rs41579631 on BTA16. For eight significant SNPs (rs29024589 on BTA1, both SNPs on BTA4, both SNPs on BTA5, both SNPs on BTA13, and rs41579631 on BTA16), significant allelic heterogeneity was detected (Table 1).

## Discussion

Summarizing the obtained results, in general, heterogeneity on a genome-wide level between breeds was observed; however, it does not explain the differences in GWAS results. Inter-breed differences in SNP effect significance at some of the 15 positions can be explained by inter-breed differences in LD structure (BTA1, BTA5, BTA13, and BTA16) indicated by high values of the *S* statistics and/or by significant allelic heterogeneity (BTA1, BTA4, BTA5, BTA13, and BTA16). Polymorphisms on BTA6 (marking *SORCS2*), BTA14 (marking *TMEM74* and *EMC2*), and BTA24 (marking *CCDC178* and *KLHL14*) are good candidates for Braunvieh-specific associations. Although the number of published studies related to GWAS for hoof disorders is very limited, their common feature is the lack of overlap in significant results both between and even within the studies. Similarly to our study, Wu et al. (2016) applied the same GWAS model to feet and leg disorders in three breeds and, depending on breed, identified different significant regions between Danish Red and Danish Holstein, while no significance was observed in Jersey. In addition, earlier, van der Spek et al. (2015) found a very low overlap in significance while analyzing a cow dataset and a bull dataset ascertained from the same population of Holstein-Friesian cattle, with only three SNPs in bulls overlapped with 94 SNPs significant for claw disorders in cows. Vargas et al. (2018) found no overlap between significant regions defined for binary and categorical feet and leg classification scores in Nelore breed. In humans, Coram et al. (2013) reported a similar result regarding loci determining concentration of lipids in blood, where many differences between populations were due to allele frequencies at the candidate SNPs. In our study, we also observed no overlap in significance between Braunvieh and Fleckvieh. The potential basis of this phenomenon is either of a statistical nature—type I/type II errors due to limited sample size, or of a genetic nature—genetic heterogeneity in the susceptibility to hoof diseases between breeds. Exploring the statistical perspective, recently, Suchocki et al. (2020) reported the results of GWAS using a multi-SNP model for the same dataset. For total number of hoof disorders, they found only two significant polymorphisms located on chromosomes 7 and 14. The positions of significant SNPs do not correspond with those detected using the single-SNP model from our study. Different results between single- and multi-SNP models suggested that each approach is capable of uncovering a different subset of QTL. The recommended solution for such situation is a meta-analysis, which allows for taking into account all former results for a given group of traits.

**FIGURE 4:**
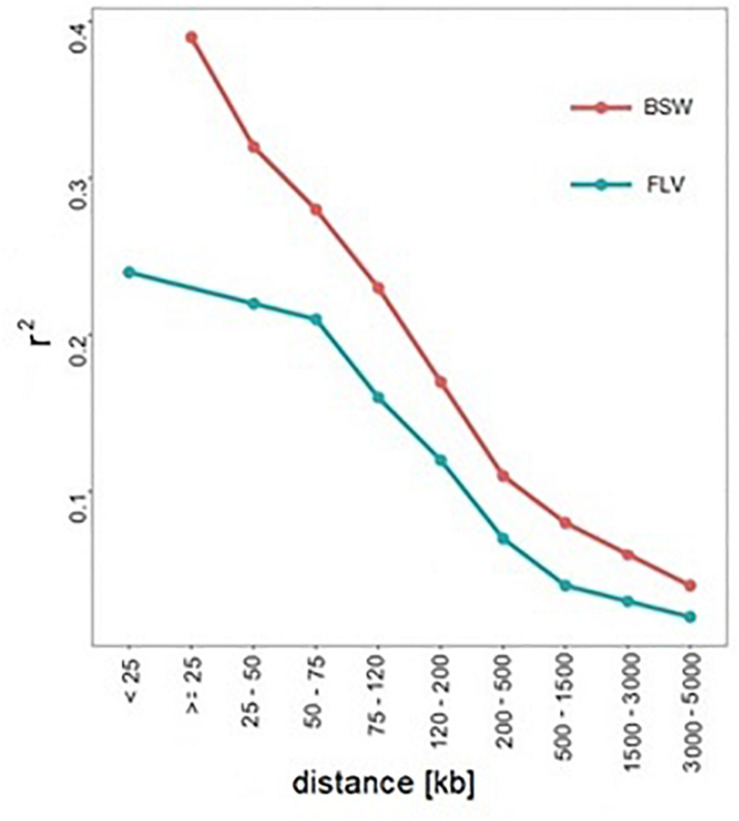
Genome-wide LD decay (represented by *r*^2^) in Braunvieh and Fleckvieh.

From the genetic perspective, Coram et al. (2013) pointed out at the presence of population-specific significant loci, which, as in our study, can be explained by population-specific selection pressure. Another postulated cause of heterogeneity [see, e.g., An and Claudianos (2016) for their discussion on autism disorder] pointed out at different causal mutations within the common metabolic pathways. Similarly, Wu et al. (2016) in the context of hoof and leg disorders in cattle hypothesized that the breed-specific significance hits represent relatively novel mutations, which occurred after breed separation. The third cause of heterogeneity is differences in genetic architecture between breeds, which are manifested by genome-wide (Figure 4) or local differences in LD, which were detected in our study within some of the regions harboring SNPs significant in Braunvieh. Differences in LD patterns were considered in the context of heterogeneity detected between human populations (Vargas et al., 2018).

## Conclusion

Based on the current dataset, it is not possible to unequivocally confirm/exclude the hypothesis of genetic heterogeneity in the susceptibility to hoof disorders between Fleckvieh and Braunvieh. The rationales against the hypothesis comprise the following: (i) limited power of detection of true associations if the effect size is not large and therefore high rate of spurious associations among detected SNP; (ii) differences in the length of LD blocks, which imply differences in power of detecting the associations; and (iii) differences in linkage phase between breeds, which may hamper the detection of causal sites in Fleckvieh or Braunvieh, based on the available SNP panel. On the other hand, (i) different selection goals defined for the breeds—toward dairy production in the case of Braunvieh and toward beef production in the case of Fleckvieh, (ii) no significant allelic heterogeneity, and (iii) no systematic differences in LD structure between the breeds remain in favor of the heterogeneity hypothesis at the significant sites on BTA6, BTA14, and BTA24.

The dataset available for the analysis comprises only common SNPs selected for a commercial microarray, so that we can explore only associations and not the causal mutations; therefore, a final verification of the above hypothesis would require a higher resolution of genomic data. However, if the total number of hoof disorders is genetically a different trait in Braunvieh and in Fleckvieh, this should be taken into account in selection strategies and in genomic evaluation programs.

## Data Availability Statement

The data analyzed in this study is subject to the following licenses/restrictions: The datasets analyzed for this study are available from the ZuchtData EDV-Dienstleistungen GmbH upon request. Requests to access these datasets should be directed to egger-danner@zuchtdata.at.

## Author Contributions

BK-S edited data and performed GWAS and heterogeneity analysis. TS edited data and performed some statistical analyses. CE-D contributed to writing of the manuscript. HS edited raw data and contributed to writing of the manuscript. MF calculated pairwise linkage disequilibrium. JS provided the funding and concept of the study, performed some statistical analyses, and significantly contributed to writing of the manuscript. All authors read and approved the final manuscript.

## Conflict of Interest

CE-D and HS were employed by the company ZuchtData EDV-Dienstleistungen GmbH. The remaining authors declare that the research was conducted in the absence of any commercial or financial relationships that could be construed as a potential conflict of interest.

## Footnotes

1 www.informatics.jax.org

2 www.mousephenotype.org

3 zfin.org

4 www.orpha.net

5 decipher.sanger.ac.uk
